# The folate-coupled enzyme MTHFD2 is a nuclear protein and promotes cell proliferation

**DOI:** 10.1038/srep15029

**Published:** 2015-10-13

**Authors:** Nina Gustafsson Sheppard, Lisa Jarl, Diana Mahadessian, Laura Strittmatter, Angelika Schmidt, Nikhil Madhusudan, Jesper Tegnér, Emma K. Lundberg, Anna Asplund, Mohit Jain, Roland Nilsson

**Affiliations:** 1 Unit of Computational Medicine, Department of Medicine, Karolinska Institutet, Stockholm, Sweden; 2 Center for Molecular Medicine, Karolinska Institutet, Stockholm, Sweden; 3 Science for Life Laboratory, Division of Translational Medicine and Chemical Biology, Department of Medical Biochemistry and Biophysics, Karolinska Institute, Stockholm, Sweden; 4 Science for Life Laboratory, Royal Institute of Technology, Solna, Sweden; 5 Department of Molecular Biology and the Center for Human Genetic Research, Massachusetts General Hospital, Boston, Massachusetts 02114, USA; 6 Department of Immunology, Genetics and Pathology, Science for Life Laboratory, Uppsala University, Uppsala, Sweden; 7 Department of Medicine, Institute for Metabolomics Medicine, University of California, San Diego, USA

## Abstract

Folate metabolism is central to cell proliferation and a target of commonly used cancer chemotherapeutics. In particular, the mitochondrial folate-coupled metabolism is thought to be important for proliferating cancer cells. The enzyme MTHFD2 in this pathway is highly expressed in human tumors and broadly required for survival of cancer cells. Although the enzymatic activity of the MTHFD2 protein is well understood, little is known about its larger role in cancer cell biology. We here report that MTHFD2 is co-expressed with two distinct gene sets, representing amino acid metabolism and cell proliferation, respectively. Consistent with a role for MTHFD2 in cell proliferation, MTHFD2 expression was repressed in cells rendered quiescent by deprivation of growth signals (serum) and rapidly re-induced by serum stimulation. Overexpression of MTHFD2 alone was sufficient to promote cell proliferation independent of its dehydrogenase activity, even during growth restriction. In addition to its known mitochondrial localization, we found MTHFD2 to have a nuclear localization and co-localize with DNA replication sites. These findings suggest a previously unknown role for MTHFD2 in cancer cell proliferation, adding to its known function in mitochondrial folate metabolism.

Cancer cells display a divergent mode of metabolism, featuring rapid glycolysis as well as anabolic processes such as synthesis of amino acids, nucleotides and lipids to support rapid cell proliferation^1^. In particular, synthesis of one-carbon units carried by the tetrahydrofolate (THF) cofactor is important for proliferating cells, required for nucleotide synthesis and methylation reactions^2^. Consequently, enzymes in the folate metabolism are targeted by anti-cancer drugs such as methotrexate and 5-fluorouracil. Folate metabolism is compartmentalized, with dual pathways present in the cytosol, nucleus and mitochondria (reviewed in^2^,^3^). The mitochondrial pathway consists of the glycine-synthesizing enzyme serine hydroxylmethyltransferase (SHMT2) and the bifunctional methylenetetrahydrofolate dehydrogenase/cyclohydrolase (MTHFD2), MTHFD2L and the monofunctional C1-tetrahydrofolate synthase (MTHFD1L), which serve to oxidize the 1-carbon unit and recycle the folate cofactor needed by SHMT2. This pathway is thought to be the main route of synthesis of glycine as well as 1-carbon units required by proliferating cells^4^,^5^, and expression of the genes in this pathway correlates with the proliferation rate across a variety of cancer cell lines^6^.

Within the mitochondrial folate pathway, the MTHFD2 enzyme is of special interest in cancer research for several reasons. A meta-analysis of gene expression data showed that MTHFD2 was one of the most consistently overexpressed mRNAs genome-wide across 19 different tumor types, and the MTHFD2 protein is specifically expressed in transformed cells but not the stroma surrounding the tumours^7^. Targeting MTHFD2 by RNAi impairs proliferation in a variety of cancer cell lines, independent of tissue of origin^7^, and decreases invasion and migration in breast cancer cell lines^8^,^9^. MTHFD2 is expressed in fetal cells and transformed cell lines, but is low or absent in most adult tissues and cell types^10^,^11^. Altogether, MTHFD2 is an attractive candidate drug target in cancer.

Although the enzymatic activity of the MTHFD2 protein is well understood^2^, little is known about its larger role in cancer cell biology. Human cancer cells depleted of MTHFD2 exhibit substantial cell death within 48 hours that cannot be rescued by glycine or formate, the products of the mitochondrial pathway^7^, suggesting that other functions besides the enzymatic activity may be important. Also, recent work suggests that the mitochondrial pathway has a role in maintaining NADPH levels as well^12^. Here, we report that MTHFD2 is present in the cell nucleus and is found at sites of DNA synthesis, and that MTHFD2 overexpression drives cell proliferation in a manner independent of the enzyme’s dehydrogenase activity.

## Results

### MTHFD2 is expressed in distinct and proliferative contexts

**Figure 1:**
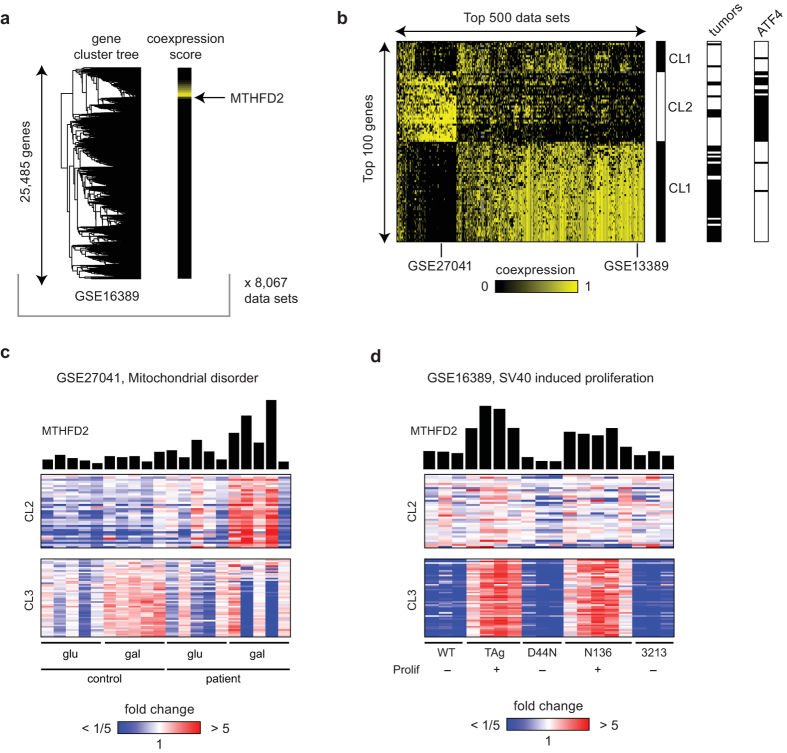
Coexpression analysis. (**a**) Example cluster tree (left) with MTHFD2 coexpresision score derived from the tree indicated for each gene (right). **(b)** Matrix of coexpression scores as in (**a**), for top scoring data sets and genes. **(c)** An example data set exhibiting coexpression between MTHFD2 and the “amino acid” gene cluster. **(d)** An example data set exhibiting coexpression between MTHFD2 and the “proliferation” gene cluster.

To search for alternative functions of the MTHFD2 protein, we performed a computational screen for genes frequently co-expressed with MTHFD2, reasoning that such genes might point to pathways or cellular processes of interest. We searched a compendium of more than 8,000 human, mouse and rat microarray data sets, and for each of these data sets, scored all genes for coexpression with MTHFD2 by measuring its distance from MTHFD2 in the cluster tree obtained by hierarchical clustering (Fig. 1a). We then summarized these scores across all data sets to identify genes that are frequently co-expressed with MTHFD2, in many independent experiments (see Methods for details).

Interestingly, the top-scoring genes separated into three clusters, which show co-expression with MTHFD2 in two distinct groups of datasets, (Fig. 1b). Cluster 1 contains genes involved in ribosome biogenesis and also nucleotide salvage. Cluster 2 includes the enzymes of the mitochondria folate pathway (SHMT2, MTHFD1L), *de novo* serine synthesis (PHGDH, PSAT1, PSPH), other amino acid-synthesizing enzymes and amino acid transporters, as well as a number of tRNA synthetases and translation factors (Table S1). This cluster closely resembles the transcriptional program initiated by the ATF4 transcription factor: MTHFD2 itself and 31/35 (88%) of the genes in cluster 2 are ATF4 targets (Fig. 1b). ATF4 is thought to mediate the “amino acid starvation” response^13^, which is initiated upon translation (ER) stress and mitochondrial dysfunction, and is also observed in some cancers^14^. An example data set exhibiting this co-expression pattern, identified by our analysis, represents fibroblasts from patients with mitochondrial disease^15^. Here, MTHFD2 and the cluster 2 were upregulated during mitochondrial stress induced by growth on a non-fermentable sugar (Fig. 1c).

In contrast, cluster 3 consisted of genes that are involved in cell cycle progression, specifically in the committed S, G2, and M phases (Table S1). Many of these genes are nuclear proteins, involved in cell cycle regulation and mitotic checkpoints. An example dataset exhibiting this co-expression pattern is shown in Fig. 1d; here MTHFD2 and cluster 3 is induced in cells driven to proliferate by the SV40 Large T antigen^16^, but cluster 2 is not. Interestingly, the genes of cluster 3 are frequently over-expressed in human tumors (Fig. 1b), as is MTHFD2^7^, but those of cluster 2 are generally not (Fig 1b). These findings raise the hypothesis that MTHFD2 may play a role in cell proliferation distinct from its function in amino acid metabolism and the ATF4 response, which may be relevant to its molecular function in cancer cells.

### MTHFD2 is rapidly induced by growth factor stimulation

**Figure 2:**
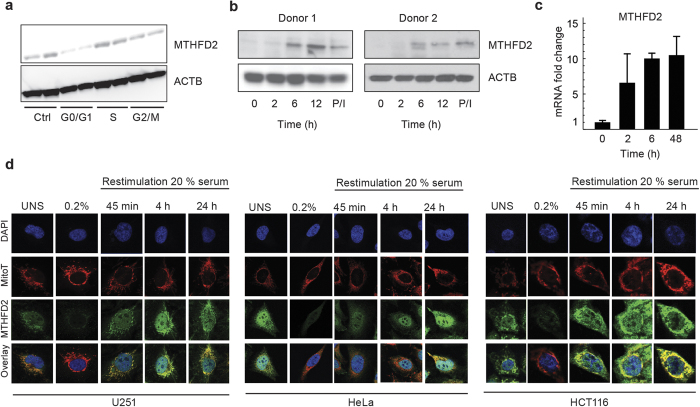
MTHFD2 is upregulated during proliferation (**a**) Immunoblot analysis of MTHFD2 in asynchronous, G0/G1, S or M phase arrested HeLa cells. β-Actin, ACTB, served as loading control. (**b**) Cropped immunoblot analysis of MTHFD2 in resting or activated T cells (anti-CD3/CD28 for indicated time periods in hours; or by PMA/ionomycin (P/I). Beta-Actin (ACTB) served as loading control. (**c**) mRNA levels of MTHFD2 in resting or activated T cells. (**d**) HCT-116, U-251 or HeLa cells in 5% serum or serum-starved in 0.2% serum for 48 hours and then treated with 20% serum for indicated time periods. Cells were then fixed, permeabilized and stained for MTHFD2, DNA (DAPI) and mitochondria (MitoT). One representative picture for each condition is shown.

The above results led us to investigate more closely the role of MTHFD2 in cell proliferation. MTHFD2 protein levels were similar in unsynchronized cells, in cells arrested in S phase by thymidine block and in cells arrested in G_2_/M phase by nocodazole (Fig. 2a, Supplementary Fig. 1a), suggesting that MTHFD2 is expressed throughout the cell cycle. Also, while depletion of MTHFD2 by siRNA causes cell death^7^, distribution between cell cycle phases among the surviving cells was not altered (Supplementary Fig. 1b). In contrast, cells deprived of growth factors by serum starvation had substantially reduced MTHFD2 protein levels (Fig. 2a). Hence, MTHFD2 appears to be expressed throughout the cell cycle in proliferating cells, but is suppressed during growth factor limitation.

To further study the response of MTHFD2 in the early transition from quiescence to proliferation in non-transformed cells, we measured MTHFD2 protein levels in 2 sets of primary human CD4+CD25- T cells from two independent healthy donors upon activation by antibodies against CD3 and CD28 or by PMA and ionomycin. MTHFD2 protein was low in quiescent T cells but increased rapidly upon stimulation, starting between 2 and 6 hours (Fig. 2b). Re-analysis of microarray data from a similar experiment on CD4^+^ T cells^17^ showed that MTHFD2 mRNA was induced already at 2 hours (Fig. 2c). Thus the increase of MTHFD2 mRNA precedes the increase in MTHFD2 protein levels during the early transition from quiescence to proliferation.

To study the serum response of MTHFD2 protein in transformed cell lines with better time resolution, we monitored MTHFD2 expression in single cells using confocal microscopy. Again, all cell lines reduced MTHFD2 expression when deprived of serum (Fig. 2d). Upon re-stimulation with serum, MTHFD2 protein levels in the mitochondria in single cells increased markedly already after 45 minutes, and completely recovered after 4 hours, thereafter maintaining stable expression levels (Fig. 2d, Supplementary Fig. 1c). Taken together, these data indicate that the mitochondrial MTHFD2 protein expression is rapidly induced in response to growth factor stimulation, in multiple cell types.

### MTHFD2 drives cancer cell proliferation independent of its dehydrogenase activity

**Figure 3:**
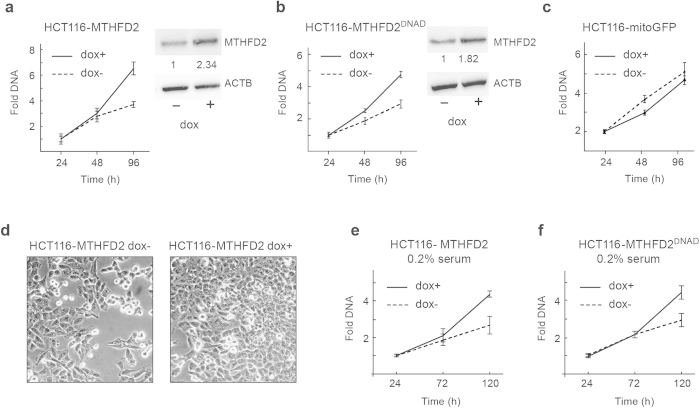
MTHFD2 drives cancer cell proliferation independently of its enzymatic activity. (**a**–**c**) HCT-116 MTFHD2, HCT-116 MTHFD2ΔNAD or HCT-116 GFP cell lines cultured in medium containing 5% serum were treated +/− 1 μg/ml doxycycline and CyQuant assays of proliferation were carried out at indicated time points. Error bars are standard deviations. *P < 0.05. MTHFD2 expression was assayed by immunoblot analysis, β-Actin, ACTB, served as loading control, the images in the figure are cropped. Numbers indicate fold induction of MTHFD2 upon DOX treatment. (**d**) Light microscopy images of conditions as in (**a**) at time-point 96 hrs. (**e**,**f**) HCT-116 MTFHD2 or HCT-116 MTHFD2Δ^NAD^ cell lines were treated +/− 1 μg/ml doxycycline for 48 hours to induce MTHFD2 or MTHFD2ΔNAD expression and then serum starved in 0.2% serum. CyQuant assays of proliferation were carried out at indicated time points. Error bars are standard deviations. *P < 0.05.

Given that MTHFD2 responds rapidly to mitogenic stimuli, we next asked whether the protein can itself promote cell proliferation, and if this role is independent of its enzymatic function. We generated HCT-116 cell lines with doxycycline-inducible expression of either the wild-type MTHFD2 protein or a mutant MTHFD2^ΔNAD^, which lacks the methylene-THF dehydrogenase activity due to a mutation R204A in the NAD-binding site^18^. Lack of dehydrogenase activity was verified by enzymatic assay of purified recombinant protein (MTHFD2 658+/−71 nmol/min/mg; MTHFD2^ΔNAD^ undetectable; n = 3). Induction of either MTHFD2 (Fig. 3a) or MTHFD2^ΔNAD^ (Fig. 3b) resulted in increased cell proliferation, whereas induction of a control protein (mito-GFP) expression did not (Fig. 3c). Cell cultures also reached confluence more rapidly after induction of MTHFD2 (Fig. 3d). Hence, the MTHFD2 protein is capable of driving cancer cell proliferation independently of its dehydrogenase activity.

To investigate whether expression of MTHFD2 override growth factor limitation, we induced MTHFD2 expression in HCT-116 cells deprived of serum (0.2% serum). Induction of either MTHFD2 (Fig. 3e) or MTHFD2^ΔNAD^ (Fig. 3f) again increased proliferation in these cultures, suggesting that a non-enzymatic function of the MTHFD2 protein can override growth factor limitation, a hallmark of transformed cells^19^.

### MTHFD2 is present in the cell nucleus

**Figure 4:**
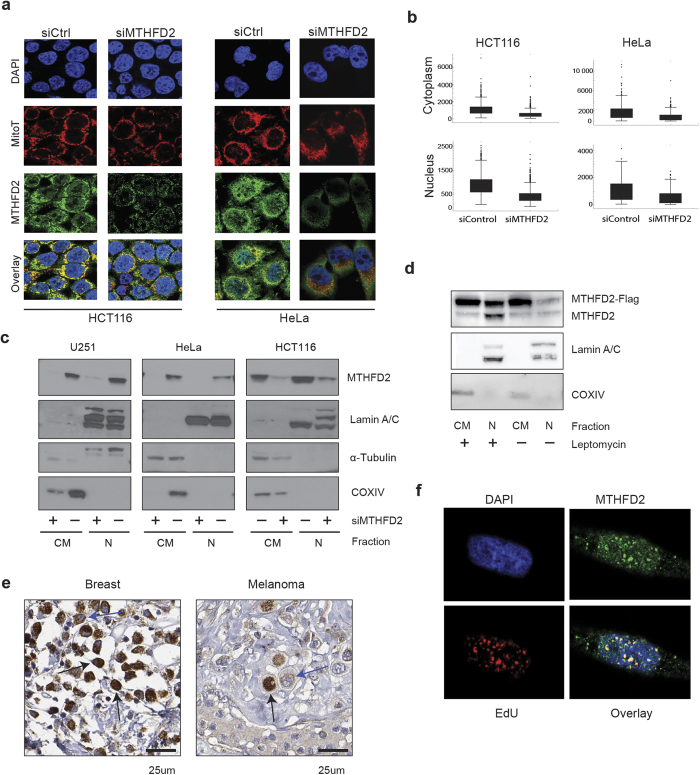
MTHFD2 is present in the cell nucleus and localizes to DNA synthesis sites. (**a**) HCT-116 or HeLa cells were transfected with 10 nM siMTHFD2 or siControl for 48 hours, fixed, permeabilised and stained for MTHFD2, DNA (DAPI) and mitochondria (MitoT). A representative image for each condition is shown. (**b**) Boxplot diagram showing quantification of nuclear and cytoplasmic localization of MTHFD2 at each condition as described in Materials and Methods. The fluorescence intensity was calculated for at least 200 cells per condition and per cell line. (**c**,**d**) Cropped immunoblot analysis of MTHFD2 in nuclear and cytosolic fractions. Lamin A/C was used as nuclear marker, α-Tubulin as cytosolic marker and COXIV as a mitochondrial marker to exclude mitochondrial contamination of nuclear fractions. (**c**) HCT-116, U-251 and HeLa cells transfected with MTHFD2 siRNA (+) or control siRNA (−). (**d**) HCT-116 cells transfected with 1 μg MTHFD2-FLAG plasmid for 24 hours and then treated +/− 10 ng/mL Leptomycin B for 12 hours. (**e**) MTHFD2 protein expression, as assessed using immunohistochemistry, in tumor cells of breast cancer and melanoma. Nuclear (black) and mitochondrial (blue) positivity indicated with arrows (**f**) U-251 cells were treated with EdU for 45 min prior to fixation and EdU detection using the Click-iT detection kit and MTFHD2 antibody staining. Pearson’s correlation coefficient (PCC) was calculated for correlation between EdU and MTHFD2 based on a pixel-by-pixel analysis in CellProfiler.

In our confocal imaging studies of serum-stimulated cells we consistently observed a nuclear localization of MTHFD2, besides the expected mitochondrial signal, and this nuclear signal was responsive to serum stimulation as well (Fig. 2d). MTHFD2 protein levels were reduced in both nuclei and mitochondria by siRNA targeting MTHFD2, indicating that the immunofluorescence antibody was specific (Fig. 4a,b). In addition, a re-analysis of proteomic data sets from isolated nuclei revealed the presence of peptides matching MTHFD2 in nuclei, but not nucleoli, and in transformed or immortalized cells, but not in normal cell types (Table S2).

We next performed subcellular fractionation experiments, separating the nucleus from the remaining cytoplasm (including mitochondria), MTHFD2 was detected both in the cytoplasmic and nuclear fractions, while the nuclear fraction was free of mitochondrial contamination (Fig. 4c). MTHFD2 siRNA again depleted MTHFD2 at 48 hours in both the cytoplasmic and nuclear fractions (Fig. 4c). We also expressed a FLAG-tagged MTHFD2 protein, and detected this protein in the nuclear fraction as well (Fig. 4d). Moreover, both endogenous MTHFD2 and MTHFD2-FLAG in the nuclear fraction increased when nuclear protein export (exportin) was inhibited by Leptomycin B treatment^20^ (Fig. 4d), suggesting that MTHFD2 protein is trafficking across the nuclear membrane.

Finally, we assessed whether nuclear localization of MTHFD2 also occurs in cancer cells in human tumors. By immunohistochemical staining for MTHFD2, we found nuclear-positive cells in a subset of breast tumor and melanoma cells (Fig. 4e). Taken together, these results demonstrate that the MTHFD2 enzyme is present in both the mitochondria and the nucleus of cancer cells.

### MTHFD2 is associated with newly synthesized DNA

In confocal imaging, we frequently observed a dot-like pattern of MTHFD2 in nucleus. A number of nuclear substructures are known that are consistent with this pattern^21^; we tested but could not detect any co-localization between MTHFD2 and PML bodies, nor with Cajal bodies (data not shown). Since other folate–metabolizing enzymes have previously been shown to be present in the nucleus and localize to DNA replication sites^22^,^23^, we hypothesized that these dots might be DNA replication sites. To test this, cells were incubated with 5-ethynyl-2-deoxyuridine (EdU) to label newly synthesized DNA. Confocal microscopy revealed a clear correlation between MTHFD2 and EdU staining (Fig. 4f), thus MTHFD2 localizes to newly synthesized DNA.

## Discussion

Our results establish that MTHFD2, previously known as a mitochondrial enzyme, has a “moonlighting” role in the nucleus as well. A number of cancer-associated metabolic enzymes have previously been found to have non-enzymatic functions in the nucleus. These moonlighting enzymes are often specific isoforms, encoded either by a paralogous gene or a splice variant, and are highly expressed by transformed cells and by embryonic or undifferentiated cell types. In glycolysis, this pattern holds true for the M2 splice variant of muscle-type pyruvate kinase (PKM2); for hexokinase 2 (HK2); for aldolase A (ALDOA): and for the regulatory enzyme PFKFB3^24^. A variety of nuclear functions have been proposed for these proteins, including DNA repair, apoptotic signaling and transcriptional effects, but overall they appear to support cell proliferation and suppress apoptosis^24^, as would be expected of proteins overexpressed in cancer cells. Whether these moonlighting functions, or the classically known metabolic activities of these proteins, or both, underlie the prevalence of these enzymes in tumors is a pressing unresolved question.

MTHFD2 is an isoform of methylene-THF dehydrogenase/cyclohydrolase which is highly expressed in embryonic cells and tumors, while the paralogous enzyme MTHFD2L is thought to be expressed by adult, differentiated cell types^25^. Previously thought to be only mitochondrial, our data shows for the first time that MTHFD2 is present in the nucleus as well. While previous subcellular fractionation studies concluded MTHFD2 to be mitochondrial, the reported experiments did not analyze a nuclear fraction^26^ or analyzed MTHFD2 localization by confocal microscopy. We further confirm presence of MTHFD2 in the nucleus in tumor samples and future experiments will address the clinical relevance of nuclear MTFHD2.

The precise function of MTHFD2 in the nucleus is not yet clear. We found MTHFD2 to co-localize with newly synthesized DNA, and we also noted that of the 50 genes in cluster 3 co-expressed with MTHFD2 (Fig. 1b), 19 genes are known to be involved in DNA synthesis or repair (Supplementary Table 1), suggesting a role for MTHFD2 in deoxynucleotide metabolism. This has previously been reported for other folate-metabolizing enzymes: CH_2_-THF for nuclear thymidylate synthesis can be obtained from serine via SHMT1/2α^27^, or from formate by MTHFD1, which recently was found to be present in the nucleus as well^28^. However, MTHFD2 lacks CHO-THF synthase activity and therefore cannot utilize formate as a source of CH2-THF; and because MTHFD2 is NAD-dependent, it is thought to operate in the direction of oxidizing CH_2_-THF to CHO-THF^2^, which theoretically would oppose CH_2_-THF (and therefore thymidylate) synthesis if enzymatically active in the nucleus. To our knowledge, there is no known reaction in the nucleus utilizing the CHO-THF that would be formed. Moreover, our computational analysis indicates that MTHFD2 is coexpressed with a cluster of predominantly nuclear genes important for cell cycle progression, which is distinct from the cluster containing the related metabolic enzymes such SHMT2 and MTHFD1L. These considerations lead us to speculate that nuclear MTHFD2 might have a non-enzymatic function, and that this function may be responsible for the observed increase in culture growth upon MTHFD2 overexpression. However, we have not yet been able to test this hypothesis; attempts to generate a nuclear-restricted MTHFD2 protein by removing the mitochondrial targeting peptide failed due to lack of protein expression. Additional studies on the precise role of MTHFD2 in the nucleus are clearly needed.

Remarkably, overexpression of MTHFD2 alone was sufficient to increase cell proliferation, and this effect was retained in a mutant protein lacking dehydrogenase activity, demonstrating that it is unrelated to the known metabolic role of MTHFD2 in the mitochondria. Growth and proliferation requires synthesis of a multitude of components that constitute biomass, and increasing a single metabolic enzyme is unlikely to affect this complex process. Rather, proteins that single-handedly affect cell growth are usually involved in “decision-making” signaling processes, raising the possibility that the non-enzymatic function of MTHFD2 is involved in signaling as well. Also, the observation that MTHFD2 promotes proliferation also in serum-deprived cells might indicate that the protein somehow overrides lack of growth signaling. However, there are many mechanisms by which a protein can affect overall culture growth, and further work is needed to provide mechanistic insight into how the MTHFD2 protein influences cancer cell proliferation.

In summary, this study demonstrates that MTFHD2 has the potential to drive proliferation independent of its dehydrogenase activity and co-localizes to DNA synthesis sites in the nucleus. This new role for MTHFD2 adds to its known mitochondrial function that provides one-carbon units for nucleotide synthesis and other methylation reactions, and further implicates MTHFD2 as a key protein for cancer cell proliferation.

## Materials and Methods

### Coexpression analysis

Gene expression data set for the coexpression analysis were retrieved from the NCBI GEO in March 2012^29^. 8,097 data sets from 44 Affymetrix microarray platforms interrogating human, mouse and rat mRNAs were used. Data sets were de-logged when needed but otherwise no additional normalization was performed. Our analysis was centered on human NCBI Entrez GeneIDs; in cases where multiple probe sets target matched one GeneID, the probe set with highest median expression across all arrays in the collection was selected. Orthologous genes in mouse and rat were mapped using homology tables from the Mouse Genome Database^30^.

For each data set, all genes were clustered using the hierarchical (agglomerative) clustering method with 1 minus the Pearson correlation as distance measure and average linkage, resulting in one cluster tree per data set. To quantify co-expression with MTHDFD2 in a given data set, for each gene X, a co-expression score *x* was defined as 1 minus (total number of genes on array)/(number of genes in cluster) for the smallest cluster containing both X and MTHFD2, so that genes clustering near MTHFD2 have a score *x* close to 1 (Fig. 1a). To focus on the strongest co-expression signals, we then applied the transform *z *= (*x* − *c*)/(1 − *c*), *x *> *c*, or 0 otherwise (mapping scores in [c, 1] onto [0, 1]), with *c *= 0.95. These scores *z* are depicted in Fig. 1b.

An integrated gene score *y* was defined as the sum of *z* over all data sets and used to select top genes in Fig. 1b. Top data sets were then selected by their similarity to the integrated gene scores, defined as the scalar product *sum_i x*_*i*_ . *y*_*i*_ . The top 500 data sets by this measure are shown in Fig. 1b. Throughout, missing values (genes not present in an array data set) were excluded from these calculations, and are indicated in gray in Fig. 1. The clusters depicted in Fig. 1b were manually delimited from a hierarchical clustering tree.

### Cell culture and reagents

HCT-116 colon cancer and U-251 glioblastoma cell lines were obtained from the National Cancer Institute. HeLa cervical cancer cell line was obtained from the European Collection of Cell Cultures. Cell lines were cultured in RPMI-1640 medium (Life Technologies) supplemented with 5% fetal bovine serum (FBS, Life Technologies) and 1% Penicillin/Streptomycin (Pen Strep, Gibco Life Technologies) and maintained at 37 °C in 5% CO2.

Inducible HCT116-MTHFD2, HCT116-MTHFD2ΔNAD and HCT116-mGFP (green fluorescent protein) were generated by viral vectors, expression was driven by a Tet-On system and induced by addition of doxycycline (1 μg/mL).

### Plasmid construction

cDNA for human MTHFD2 was obtained from the Dana-Farber/Harvard Cancer Center DNA Resource Core (HsCD00040365). This was amplified via PCR to append BP sites, as well as the first 18 nt of MTHFD2 missing from the DF/HCC cDNA construct. It was introduced into pDONR221 via Gateway BP reaction (Invitrogen). Synonymous point mutations were made using Agilent QuikChange Lightning, according to the manufacturer’s protocols, to confer RNAi resistance to two shRNA hairpins (Clone ID TRCN0000036553, Clone name NM_006636.2-772s1c1, Target sequence 5′-GCAGTTGAAGAAACATACAAT-3′; and Clone ID TRCN0000036551, Clone name NM_006636.2-1067s1c1, Target sequence 5′-CGAGAAGTGCTGAAGTCTAAA-3′), and to add a stop codon, yielding the cDNA construct for MTHFD2. To generate dehydrogenase-dead MTHFD2, Agilent QuickChange Lightning was used on the previously described plasmid to mutate the Arginine in the MTHFD2 protein sequence KNVVVAGRSKN to Alanine^18^. Mitochondrial-targeted eGFP in pDONR221 was obtained from the Mootha Laboratory. All three cDNAs for overexpression were cloned into PCWPUROCV5[ccdB] via LR reaction (Invitrogen).

MTHFD2-3xFLAG was constructed by deleting the stop codon in the cDNA encoding human MTHFD2 (Origene) and then ligated into CMV14-3xFlag (Sigma Aldrich) using standard PCR-based cloning. Correct sequences were verified using DNA sequencing.

### Generation of stable cell lines

The inducible overexpression plasmids were each packaged with VSVG and pCMV-dR8.91 in 293T cells to generate lentivirus, as previously described (Jain, Science, 2012). A day before transfection, 293T cells were seeded in 6 well dishes (850,000 cells/well in 2 mL). 24 hours later, these cells were transfected with 1 μg of each inducible viral vector, 900 ng pCMV-dR8.91, and 100 ng VSVG with 6 μL Roche X-tremeGENE 9 DNA transfection reagent for each well/plasmid, according to the manufacturer’s protocol. 18 hours later, high serum (30% Sigma F6178 FBS in DMEM) media was added. Virus was harvested at 24 and 48 hours, pooled and frozen at –80 °C in single-use aliquots. Lentiviral infection was performed as previously described^6^. Stable HCT116 cell lines were generated by lentiviral infection and selection with 4 μg/mL puromycin. Overexpression of each construct was induced by addition of doxycycline (1 μg/mL).

### Transient transfections

Small interfering RNA (siRNA) oligonucleotides targeting MTHFD2 were previously validated and transfections were carried out at 10 nM as previously described^7^. Plasmid transfections were carried out 24 hours post-plating using jetPRIME according to the manufacturer’s instructions (Polyplus transfection).

### Proliferation assay

HCT116-MTHFD2, HCT116-MTHFD2ΔNAD or HCT116-mGFP transduced cell lines were treated for 48 hours with 1 μg/mL doxycycline, plated in 96 well plates and at time point for harvest; rinsed with PBS and stored at −80 °C prior to CyQuant cell proliferation assay (Life Technologies) according to manufacturer’s instructions.

### T cell isolation and culture

Buffy coats from anonymized donors were purchased from the Karolinska University Hospital according to the national ethical regulations in Sweden. Peripheral blood mononuclear cells (PBMCs) were obtained by Ficoll density gradient centrifugation, monocytes depleted by plastic adherence and platelets removed by low-speed centrifugations (200 g). CD4+CD25- T cells were obtained from PBMCs by negative isolation with magnetic beads (human CD4 T cell isolation kit, and additional depletion of CD25+ cells with CD25 microbeads) according to the manufacturer’s instructions (Miltenyi Biotec). T cells were cultured at 5% CO_2_/37 °C in serum-free X-Vivo 15 medium (Lonza) supplemented with 1% Glutamax (Life Technologies). T cells were stimulated with 5 μg/ml plate-bound anti-CD3 antibody (clone OKT3) and 1 μg/ml soluble anti-CD28 antibody (clone CD28.2; both Biolegend, LEAF grade) or with 0.5 μM ionomycin and 10 ng/ml PMA (both Sigma-Aldrich)

### Cell cycle studies

HeLa cells were plated in 6 well plates at a density of 200 000 cells per well. A G0/G1 population was generated by 48 hours serum starvation in 0.2% serum, an S phase population was achieved by 2 mM thymidine (Sigma) treatment for 18 hours and a population in the G2/M phase by 18 hours treatment with 2 mM thymidine followed by 3 hours treatment with 100 ng/mL nocodazole (Sigma). Cells were lysed in Radio-Immunoprecipitation Assay (RIPA) Buffer, protein concentration was measured by BCA protein assay kit (Pierce) and equal amounts of protein were loaded in Laemmli sample loading buffer and subjected to 4–12% SDS–PAGE followed by Western Blot analysis with antibodies against MTHFD2 (Abcam) and β-Actin (Sigma). As a control for cell cycle arrest in different phases, cells were harvested and fixed in 70% ethanol over night at 4 °C followed by staining with propidium iodide. Staining was measured using flow cytometry by the fluorescence intensity of 20,000 cells, data acquisition was done on a CyAn ADP analyzer (Beckman Coulter), and data were analyzed using ModFit Software.

### Subcellular fractionations

U-251, HCT-116 or HeLa cells were plated in 100mm dishes and subcellular fractionations were performed as previously described^31^. Briefly, cells were centrifuged for 5 minutes at 1500 rpm, then lysed in buffer A (10mM HEPES, pH 7.5, 10 mM NaCl, 0.1 mM EDTA, 0.1 mM EGTA, 1mM DTT and complete protease inhibitors (Roche)), and then incubated for 15 min on ice, 10% NP-40 was added followed by vigorous vortexing, nuclei were spun down by centrifugation for 5 minutes at 3000 rpm. The pellet was re-suspended in buffer C (20mM HEPES, pH 7.5, 420 mM NaCl, 0.1 mM EDTA, 0.1 mM EGTA, 20% glycerol, 1 mM DTT and complete protease inhibitors (Roche)) and left for 30 minutes on ice followed by 13000 rpm for 30 minutes. Subcellular fractions were dissolved in/supplemented with Laemmli sample-loading buffer and subjected to 4–12% SDS–PAGE followed by Western Blot analysis with antibodies against FLAG M2 (Sigma), MTHFD2 (Abcam), Lamin A/C, COXIV and α-Tubulin (Pierce). For experiments involving transient transfection and treatment with 10 ng/mL Leptomycin B (Sigma), cells were seeded 24 hours before transfection and allowed to recover 24 hours before leptomycin B treatment for 12 hours.

### Confocal microscopy

HCT-116, U-251 and HeLa cells were plated on coverslips (13 mm in diameter) in 12-well plates at a density of 40 000 cells per well. At indicated times post-transfection, growth medium was changed to fresh, pre-warmed medium containing 300 nM MitoTracker Deep Red FM (Life Technologies), followed by 45 min incubation at 37 °C. Cells were fixed in 3% paraformaldehyde and 5% sucrose for 15 min, followed by fixation and permeabilisation in 1:1 methanol:acetone (MeOH:Ac) for 10 min at −20 °C. Cells were further permeabilised with 0.2% NP-40 for 10 min, blocked in 5% normal goat serum (Life Technologies) and 5 mg/mL Bovine Serum Albumin (BSA) (Sigma) for 15 min. Incubation with primary antibody was done overnight at 4 °C followed by incubation with secondary antibody for one hour. The following antibodies diluted in 5 mg/mL BSA, 0.1% Tween20 in PBS were used: mouse monoclonal MTHFD2 antibody (Abcam, ab56772, 1:100), mouse monoclonal) and Alexa Flour 488 goat anti-mouse IgG (H+L) antibody (Life Technologies, A-11029, 1:500). Coverslips were mounted with ProLong Gold Antifade Reagent and stained with 4′,6-diamidino-2-phenylindole (DAPI, Life Technologies). Images were acquired in a Leica TCS SP5 confocal microscope with 40× and 63× oil immersion objectives.

For DNA synthesis studies, cells were incubated with 10 mM EdU for 45 minutes prior to fixation, EdU detection was performed using the Click-iT detection kit according to the manufacturer’s instructions (Life Technologies).

### Fluorescence Intensity Quantification

We used CellProfiler^32^ to quantify the integrated fluorescence intensity. The cell was segmented into nucleus, as defined using DAPI boundaries, and cytoplasm, as defined using MitoTracker boundaries. Fluorescence intensity for MTHFD2 was calculated for segmented nucleus and segmented cytoplasm. Graphs were plotted in Mathematica using the integrated intensity per segmented region.

### Immunohistochemistry

Tissue specimens were collected in accordance with approval from the local ethics committee (ref # Ups 02–577) and according to Swedish rules and legislation. Tissue microarrays holding samples from the different cancer types as well as from normal tissues were produced as previously described 38. Slides were deparaffinized in xylene, hydrated in graded alcohols and blocked for endogenous peroxidase for 5 min in 0.3% H2O2 diluted in 95% ethanol. Heat-induced epitope retrieval was done in a decloaking chamber (Biocare Medical) with citrate buffer at pH 6.0 for 4 min at 125 °C. Before staining, slides were immersed in wash buffer containing 0.2% Tween-20 for 15 min to avoid surface tension. Staining was performed in an Autostainer 480S instrument (Thermo Fisher Scientific) at room temperature with the following steps: Ultra V block (Thermo Fisher Scientific, no. TA-125-UB) 5 min, primary antibody 30 min, primary antibody enhancer (Thermo Fisher Scientific, no. TL-125-PB) 20 min, UltraVision LP HRP polymer (Thermo Fisher Scientific, no. TL-125-PH) 30 min, and diaminobenzidine (Thermo Fisher Scientific, no. TA-125-HDX) 5 min × 2 times. MTHFD2 primary antibody was from Abnova (mouse monoclonal, no. H00010797-M01). Between incubations, slides were rinsed in wash buffer. Slides were counter-stained with Mayer’s hematoxylin (Histolab, Gothenburg, Sweden, ref. 01820), dehydrated and mounted on cover slips using pertex (Histolab, ref. 00871.0500). Two independent observers evaluated the staining.

### Statistical analysis

All data are presented as mean ± standard deviation. For statistical comparison of two groups, unpaired, two-tailed Student’s *t*-test was used, a *P* value of < 0.05 was considered significant.

## Additional Information

**How to cite this article**: Gustafsson Sheppard, N. *et al*. The folate-coupled enzyme MTHFD2 is a nuclear protein and promotes cell proliferation. *Sci. Rep*. **5**, 15029; doi: 10.1038/srep15029 (2015).

## Supplementary Material

Supplementary Information

Supplementary Table S1
