# Supplementary material for: The folate-coupled enzyme MTHFD2 is a nuclear protein and promotes cell proliferation
Source: Sci Rep. 2015 Oct 13;5:15029. doi: 10.1038/srep15029 (PMC4602236; doi:10.1038/srep15029)
Supplement: Supplementary Information [file srep15029-s1.pdf]

The folate-coupled enzyme MTHFD2 is a nuclear protein and promotes cell proliferation

Nina Gustafsson Sheppard[1,2,3,], Lisa Jarl[1,2], Diana Mahadessian[4], Laura Strittmatter[5], Angelika Schmidt[1,2], Nikhil Madhusudan[5], , Jesper Tegnér[1,2], Emma K Lundberg[4], Anna Asplund[6], Mohit Jain[7], Roland Nilsson[1,2,*]

Table S1

Cluster 1

Symbol	Description
GCH1	GTP cyclohydrolase 1
UCK2	uridine-cytidine kinase 2
LRRC59	leucine rich repeat containing 59
EIF3J	eukaryotic translation initiation factor 3, subunit J
GNL3	guanine nucleotide binding protein-like 3 (nucleolar)
NOLC1	nucleolar and coiled-body phosphoprotein 1
DDX21	DEAD (Asp-Glu-Ala-Asp) box helicase 21
CTPS1	CTP synthase 1
GTPBP4	GTP binding protein 4
EEF1E1	eukaryotic translation elongation factor 1 epsilon 1
EIF2S1	eukaryotic translation initiation factor 2, subunit 1 alpha, 35kDa
WDR12	WD repeat domain 12
HSPD1	heat shock 60kDa protein 1 (chaperonin)
PSMD14	proteasome (prosome, macropain) 26S subunit, non-ATPase, 14
MRPL3	mitochondrial ribosomal protein L3

Amino Acid Cluster

Symbol	Description
MTHFD1L	methylenetetrahydrofolate dehydrogenase (NADP+ dependent) 1-like
BCAT1	branched chain amino-acid transaminase 1, cytosolic
HSPA9	heat shock 70kDa protein 9 (mortalin)
LARS	leucyl-tRNA synthetase
EPRS	glutamyl-prolyl-tRNA synthetase
IARS	isoleucyl-tRNA synthetase
TARS	threonyl-tRNA synthetase

IFRD1	interferon-related developmental regulator 1
PSPH	phosphoserine phosphatase
AARS	alanyl-tRNA synthetase
NARS	asparaginyl-tRNA synthetase
ATF4	activating transcription factor 4 (tax-responsive enhancer element B67)
XPOT	exportin, tRNA (nuclear export receptor for tRNAs)
EIF2S2	eukaryotic translation initiation factor 2, subunit 2 beta, 38kDa
SLC7A5	solute carrier family 7 (amino acid transporter light chain, L system), member 5
SLC7A1	solute carrier family 7 (cationic amino acid transporter, y+ system), member 1
SLC1A5	solute carrier family 1 (neutral amino acid transporter), member 5
PSAT1	phosphoserine aminotransferase 1
PHGDH	phosphoglycerate dehydrogenase
SHMT2	serine hydroxymethyltransferase 2 (mitochondrial)
EIF4EBP1	eukaryotic translation initiation factor 4E binding protein 1
MARS	methionyl-tRNA synthetase
GARS	glycyl-tRNA synthetase
CARS	cysteinyl-tRNA synthetase
ASNS	asparagine synthetase (glutamine-hydrolyzing)
YARS	tyrosyl-tRNA synthetase
SLC1A4	solute carrier family 1 (glutamate/neutral amino acid transporter), member 4
CTH	cystathionase (cystathionine gamma-lyase)
SLC7A11	solute carrier family 7 (anionic amino acid transporter light chain, xc- system), member 11
WARS	tryptophanyl-tRNA synthetase
CHAC1	ChaC, cation transport regulator homolog 1 (E. coli)
CEBPG	CCAAT/enhancer binding protein (C/EBP), gamma
TRIB3	tribbles homolog 3 (Drosophila)
SARS	seryl-tRNA synthetase
PCK2	phosphoenolpyruvate carboxykinase 2 (mitochondrial)

Cell Cycle Cluster

Symbol	Description
HAT1	histone acetyltransferase 1
USP1	ubiquitin specific peptidase 1
EZH2	enhancer of zeste homolog 2 (Drosophila)
CKS1B	CDC28 protein kinase regulatory subunit 1B
CDC6	cell division cycle 6 homolog (S. cerevisiae)
RRM1	ribonucleotide reductase M1
MCM4	minichromosome maintenance complex component 4
DBF4	DBF4 homolog (S. cerevisiae)
HMGB2	high mobility group box 2
PCNA	proliferating cell nuclear antigen
H2AFZ	H2A histone family, member Z
RFC4	replication factor C (activator 1) 4, 37kDa
MCM2	minichromosome maintenance complex component 2
FEN1	flap structure-specific endonuclease 1

MCM6	minichromosome maintenance complex component 6
SMC4	structural maintenance of chromosomes 4
TRIP13	thyroid hormone receptor interactor 13
CHEK1	checkpoint kinase 1
PLK4	polo-like kinase 4
CENPA	centromere protein A
KIAA0101	KIAA0101
RAD51	RAD51 homolog (S. cerevisiae)
DTL	denticleless E3 ubiquitin protein ligase homolog (Drosophila)
ECT2	epithelial cell transforming sequence 2 oncogene
TPX2	TPX2, microtubule-associated, homolog (Xenopus laevis)
AURKA	aurora kinase A
BIRC5	baculoviral IAP repeat containing 5
CDC20	cell division cycle 20 homolog (S. cerevisiae)
CCNB2	cyclin B2
PBK	PDZ binding kinase
RRM2	ribonucleotide reductase M2
NUSAP1	nucleolar and spindle associated protein 1
PRC1	protein regulator of cytokinesis 1
CEP55	centrosomal protein 55kDa
TOP2A	topoisomerase (DNA) II alpha 170kDa
RAD51AP1	RAD51 associated protein 1
MAD2L1	MAD2 mitotic arrest deficient-like 1 (yeast)
RACGAP1	Rac GTPase activating protein 1
HMMR	hyaluronan-mediated motility receptor (RHAMM)
CCNA2	cyclin A2
TTK	TTK protein kinase
BUB1	budding uninhibited by benzimidazoles 1 homolog (yeast)
MELK	maternal embryonic leucine zipper kinase
KIF2C	kinesin family member 2C
CCNB1	cyclin B1
CDK1	cyclin-dependent kinase 1
BUB1B	budding uninhibited by benzimidazoles 1 homolog beta (yeast)
DLGAP5	discs, large (Drosophila) homolog-associated protein 5
KIF11	kinesin family member 11
ASPM	asp (abnormal spindle) homolog, microcephaly associated (Drosophila)

Table S3

First author	Pubmed ID	Cell state	Species	Cell type	Compartment	MTHFD2
Han	20515076	Transformed	Human	HCT-116, A375	Whole nucleus	Y
Hwang	16540461	Transformed	Human	Jurkat	Whole nucleus	Y
Mulvey	23320540	Immortalized	Human	IMR90 fibroblasts	Whole nucleus	Y
Yu	19190182	Immortalized	Mouse	Immortalized	Soluble nuclear extract	Y
Andersen	15635413	Transformed	Human	HeLa	Nucleolus	N
Andersen	11790298	Transformed	Human	HeLa	Nucleolus	N
de Mateo	21630459	Normal	Human	Sperm	Whole nucleus	N
Desrivières	17514683	Immortalized	Mouse	HC11 epithelial cells	Whole nucleus	N
Dreger	11593002	Transformed	Mouse	Neuroblastoma	Nuclear envelope	N
Franklin	20807835	Normal	Mouse	Heart	Whole nucleus	N
Malmstrom	14613209	Normal	Human	Primary fibroblast	Whole nucleus	N
Tchapyjnikov	19996160	Normal	Rat	Kidney medulla	Whole nucleus	N
Turck	14730675	Transformed	Human	Caco2	Whole nucleus	N
Ying	16815949	Embryonic	Human	Fetal liver	Whole nucleus	N

Figure S1

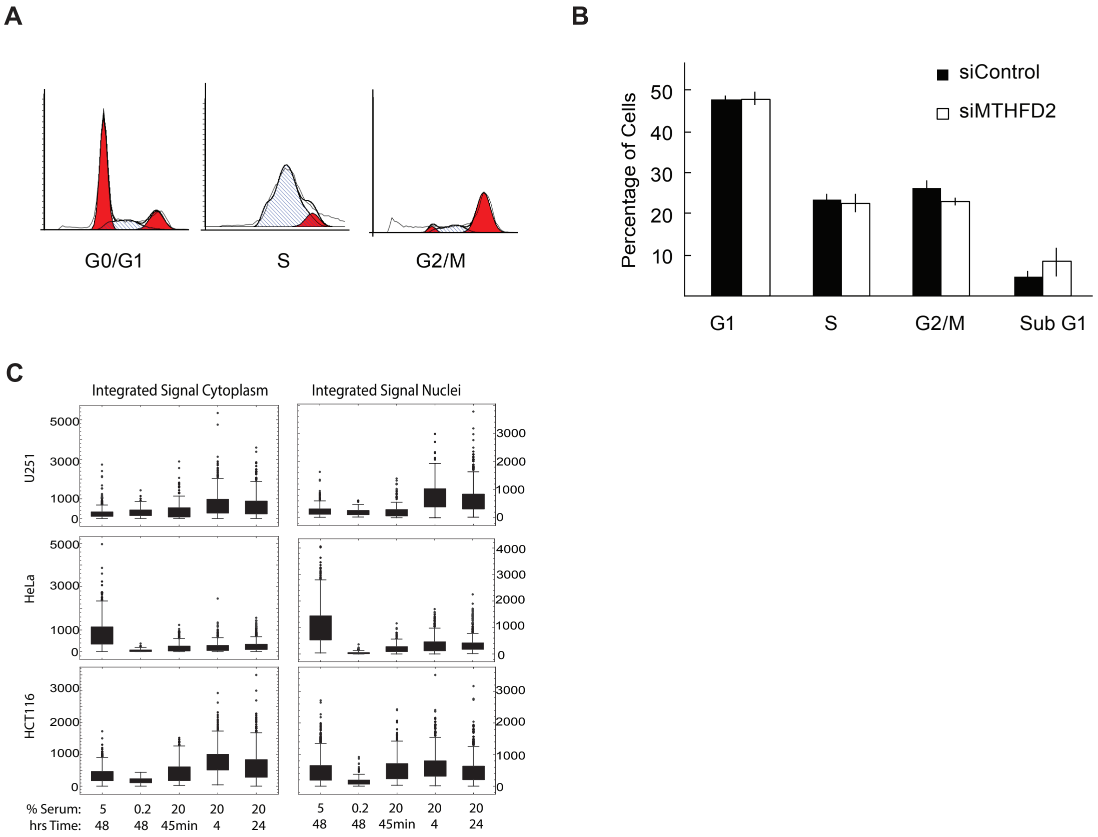

Figure S1. Cell cycle analysis. A) Flow cytometry analysis of DNA content in asynchronous, G0/G1, S or M phase arrested HeLa cells. **B)** Flow cytometry analysis of DNA content upon 72hrs of siMTFHD2 or siControl transfections in HeLa cells. **C)** Boxplot diagram showing quantification of nuclear and cytoplasmic localization of MTHFD2 at each condition, as described in Materials and Methods. The fluorescence intensity was calculated for 200-2000 cells per condition and cell line.
